# Bias of shear wave elasticity measurements in thin layer samples and a simple correction strategy

**DOI:** 10.1186/s40064-016-2937-3

**Published:** 2016-08-12

**Authors:** Jianqiang Mo, Hao Xu, Bo Qiang, Hugo Giambini, Randall Kinnick, Kai-Nan An, Shigao Chen, Zongping Luo

**Affiliations:** 1 Department of Orthopedics, The First Affiliated Hospital of Soochow University, Orthopedic Institute, Medical College, Soochow University, Suzhou, Jiangsu People’s Republic of China; 2 Basic Ultrasound Research Laboratory, Department of Physiology and Biophysics, Mayo Clinic, Rochester, MN USA; 3 Biomechanics Laboratory, Division of Orthopedic Research, Mayo Clinic, Rochester, MN USA; 4 Biomaterials and Tissue Engineering Laboratory, Division of Orthopedic research, Mayo Clinic, Rochester, MN USA; 5 Department of Radiology, Mayo Clinic, Rochester, MN USA

**Keywords:** Shear wave elastography, Ultrasound, Thin layer, Finite element method

## Abstract

Shear wave elastography (SWE) is an emerging technique for measuring biological tissue stiffness. However, the application of SWE in thin layer tissues is limited by bias due to the influence of geometry on measured shear wave speed. In this study, we investigated the bias of Young’s modulus measured by SWE in thin layer gelatin–agar phantoms, and compared the result with finite element method and Lamb wave model simulation. The result indicated that the Young’s modulus measured by SWE decreased continuously when the sample thickness decreased, and this effect was more significant for smaller thickness. We proposed a new empirical formula which can conveniently correct the bias without the need of using complicated mathematical modeling. In summary, we confirmed the nonlinear relation between thickness and Young’s modulus measured by SWE in thin layer samples, and offered a simple and practical correction strategy which is convenient for clinicians to use.

## Background

Tissue elastic property, or stiffness, is an important characteristic of biological tissues. It is determined by the cells and extracellular matrix that make up of the tissues. Therefore, pathological changes at cell or molecule level could cause characteristic elastic property changes. It is now well recognized that tissue stiffness is a useful biomarker for evaluation of many diseases. Among the multiple potential clinical applications, tissue stiffness has been extensively investigated as a biomarker for cancer diagnosis and liver fibrosis staging (Cosgrove et al. 2013). Palpation has long been used by physicians to assess tissue stiffness for making diagnosis. Ultrasound elastography initially proposed in 1990’ was a significant advancement (Ophir et al. 1991), which provides objective measurements of tissue elastic property in addition to traditional anatomical images. Since then, this method has been widely studied for diagnoses of liver fibrosis (Bavu et al. 2011; Zhao et al. 2014), breast tumor (Zhang et al. 2015; Stoian et al. 2016), skeleton muscle lesion (Hatta et al. 2015), prostate cancer (Woo et al. 2014), and others.

Two major approaches are currently used for ultrasound elastography: examination of the strain or deformation of a tissue due to an applied force (strain elastography, SE) (Ophir et al. 1991) and analysis of the propagation speed of shear waves generated by mechanical vibration or ultrasound radiation force (shear wave elastography, SWE) (Bercoff et al. 2004). SWE is often preferred over SE, because it can offer quantitative assessment and is less operator-dependent. Therefore, SWE has gained a lot of clinical attention and is currently under active research.

Shear wave elastography measures the local propagation speed of shear wave *c*_*s*_ to estimate the Young’s modulus *E* of tissue through

1 $$E = 3\rho c_{s}^{2}$$

where *ρ* is the density of tissues commonly assumed to be a constant of 1000 kg/m^3^. Equation (1) assumes local homogeneousness in an infinite medium. These assumptions are valid for large parenchymal organs such as liver or breast. In organs with thin layer structure, such as myocardium, blood vessel wall, and bladder wall, the shear wave speed is determined by the thickness of the thin layer in addition to the mechanical properties. Therefore, Eq. (1) can no longer give correct estimation of Young’s modulus of tissues within thin layers. Some studies attempted to use a Lamb wave model to describe the propagation of waves in a thin layer, so that the correct shear modulus can be solved from the measured *c*_*s*_ using the model and the thickness of the thin layer (Brum et al. 2012; Couade et al. 2010; Nguyen et al. 2011; Nenadic et al. 2011). However, this approach involves complex equations and therefore may be difficult for daily use by clinicians.

In this study, we developed a simple empirical formula to correct for the bias induced by SWE measurements in thin layer structures. The effects of the sample thickness and shear wave frequency were investigated. Results of the empirical formula were compared with the Lamb wave model, finite element method (FEM), and experimental data in phantoms of different stiffness and thickness. This method allows easy conversion of measured shear wave speed to Young’s modulus in thin layer structure of any thickness.

## Methods

### Experiments

#### Phantom preparation

**Fig. 1:**
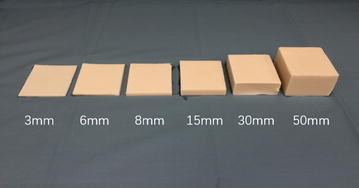
One set of gelatin–agar phantoms made for SWE experiment (three sets of phantoms were of same shape and size but different stiffness). The cross section of the phantoms was 80 × 80 mm. The thickness of the phantoms was labeled on the figure

Three sets of gelatin–agar phantom samples with different stiffness were made for SWE test (Fig. 1). Each set consisted of 6 samples with same end area (80 × 80 mm), but different thickness (3, 6, 8, 15, 30 and 50 mm respectively). The stiffness of the phantoms was controlled by the concentration of agar used. Evaporated milk (with a volume ratio of 1:1 to distilled water) was used to increase attenuation to ultrasound for more realistic simulation of real tissues. The main ingredients were gelatin (3 %) and agar (2, 2.5 and 3 % respectively). We also added 2 % potassium sorbate for preservation and 1 % cellulose to enhance ultrasound signal scattering. All ingredient proportions were measured by weight, and manufactured by Sigma-Aldrich, St Louis, MO, USA. The mixture were heated to 90 °C in a water bath to fully dissolve the gelatin and agar,and then poured into plastic molds and cured for 24 h under room temperature (23 °C).

#### Experimental setup for SWE

**Fig. 2:**
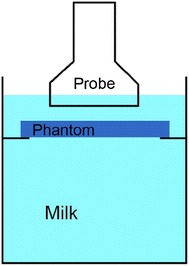
Experimental setup for SWE

SWE measurements on phantoms of different thickness were obtained. To minimize the influence of other confounding factors such as boundary conditions, we used the experimental setup shown in Fig. 2. The samples were suspended in a cylinder acrylic container (diameter 120 mm, height 200 mm) filled with evaporated milk, and the ultrasound transducer was positioned at 1 cm above the samples. Evaporated milk was used to simulate attenuation to ultrasound imposed by real tissues, so that reflection of ultrasound by the container will not introduce errors to measurements. The edge of the phantom and the transducer were fixed, so as to avoid unwanted motion which may cause artifacts on ultrasound images.

#### Shear wave elastography protocol

**Fig. 3:**
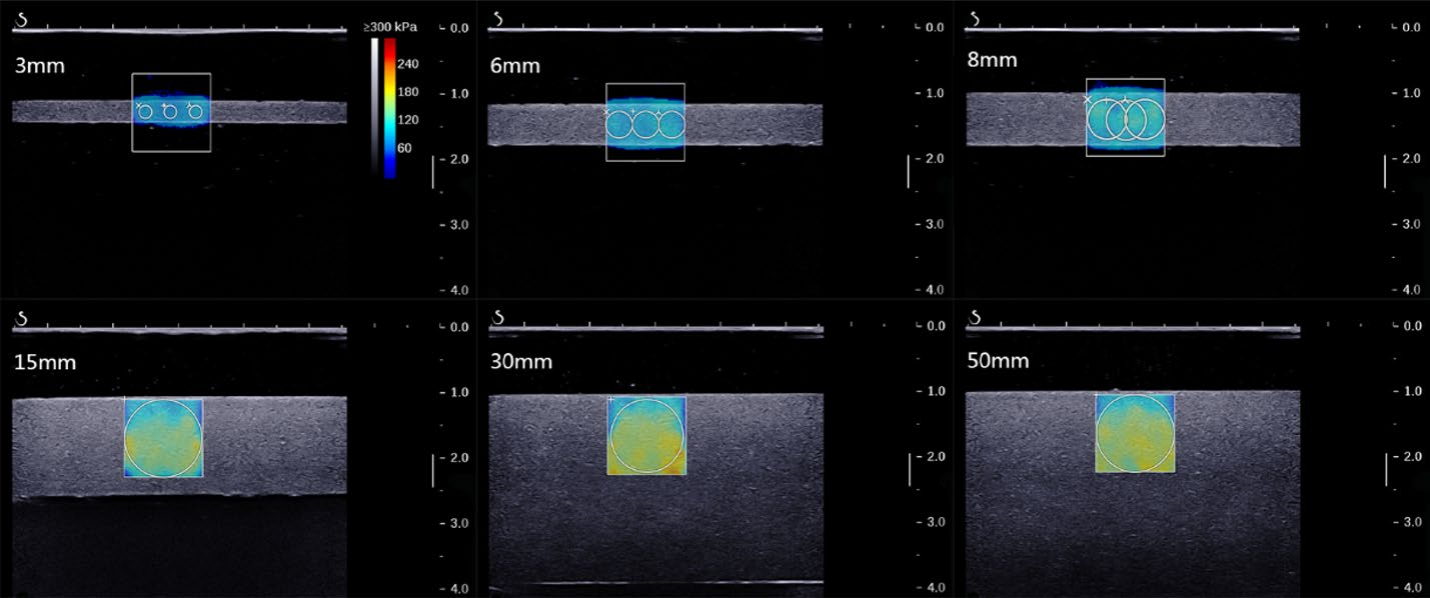
SWE images of gelatin–agar phantoms (3 % agar)

A commercial ultrasound scanner (Aixplorer, SuperSonic Imagine, France) with a SL15-4 linear array transducer was used to obtain the SWE images of the phantoms. The bandwidth of the transducer is 4–15 MHz, with a resolution of 0.3 mm in B-mode and a resolution of 1 mm in shear wave elastography mode. The settings of SWE were configured as follows: MSK (Muscular-skeletal effects) mode, elasticity range 300 kPa, opacity 50 %, auto TGC (time gain compensation). Images were captured when the shear wave speed signal was stable under SWE mode. Three different region of interests (ROI) with diameters of 2, 4 and 6 mm respectively were selected for thin phantoms (3, 6 and 8 mm). A big ROI with diameter of 11.84 mm was selected underneath the surface for thicker phantoms (15, 30 and 50 mm) (Fig. 3). Three images with good quality were obtained for each phantom. The average Young’s modulus of each phantom was calculated by averaging the mean value indicated in each ROI.

### Finite element method and the empirical formula

**Fig. 4:**
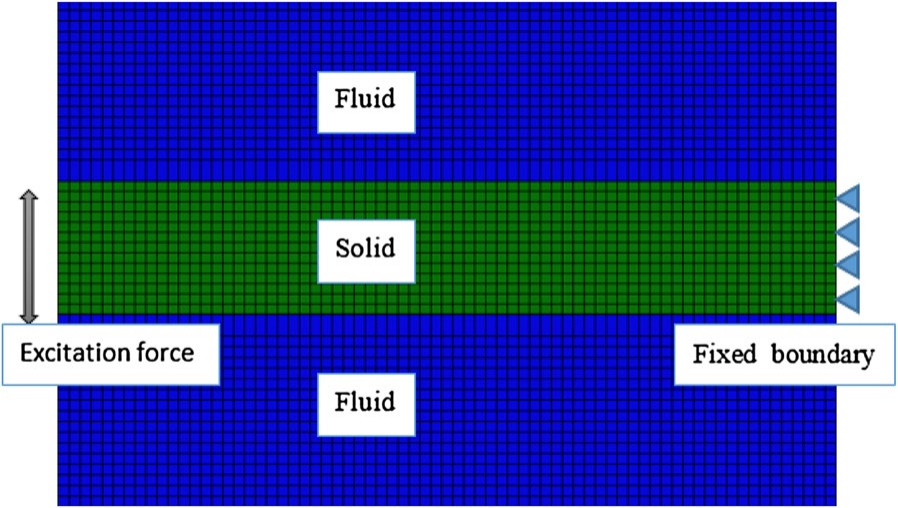
Two dimensional FEM model of the SWE experiment

A 2D FEM model of a thin-plate submerged in fluid was designed using ABAQUS 6.11 to investigate the wave propagation in the thin plate (Kanai 2005) (Fig. 4). The incompressible fluid and thin-plate were represented by axisymmetric acoustic element (ACAX4R) and axisymmetric element (CAX4R) respectively. The bulk modulus and density of the fluid were 2.2 GPa (Nenadic et al. 2010) and 1050 kg/m^3^ respectively. The Poisson’s ratio and density of the thin plate were 0.499 and 1050 kg/m^3^. The Young’s modulus of the biggest samples (50 mm) of each set was used in our simulation. A vibration stimulus of four cycle of sinusoidal wave with an amplitude of 1 μm and loading frequency of 300, 600 and 900 Hz respectively was applied throughout the entire thickness of the phantoms as a line source. The excitation was applied along the middle axis of the phantoms. A fixed boundary condition was prescribed to the lateral surface of the phantoms. Shear wave velocity was recorded with a 0.5 mm spatial resolution along a line extending away from the source in the middle of the plate. Wave speed at each thickness was calculated from the simulation data and compared with SWE measurements in phantoms of different stiffness and thickness to estimate the center frequency of shear waves in SWE measurements. An empirical formula was then derived from the results of FEM simulation to correct for measurement bias in thin layers. This empirical formula used the thickness of the layer and the “appearant” stiffness measured by SWE in the thin layer to estimate the “true” stiffness of the material. Results were then compared with experimental data in phantoms.

### Anti-symmetric Lamb wave model

The anti-symmetric Lamb wave model (Nenadic et al. 2011) describing the propagation of shear waves in a thin layer structure was used to compare with results of FEM, empirical formula, and experimental data in this study. The anti-symmetric Lamb wave dispersion equation has the following form:

2 $$\begin{aligned} & 4k_{l}^{3} \beta \cosh \left( {k_{l} h} \right)\sinh \left( {\beta h} \right) \\ & \quad = \left( {k_{s}^{2} - 2k_{l}^{2} } \right)^{2} \sinh \left( {k_{l} h} \right)\cosh \left( {\beta h} \right) + k_{s}^{4} \cosh\left( {k_{l} h} \right)\sinh\left( {\beta h} \right) \\ \end{aligned}$$

where *k*_*l*_ = *ω*/*c*_*L*_ and $k_{s} = \omega /\sqrt {{\text{G}}/\rho }$ are the Lamb wave number and shear wave number respectively, $\beta = \sqrt {k_{l}^{2} - k_{s}^{2} }$, h is the half-thickness of the sample, *ρ* is density, G is shear modulus, *c*_*L*_ is the Lamb wave velocity, ω is the angular frequency.

## Results

### Shear wave elastography

**Fig. 5:**
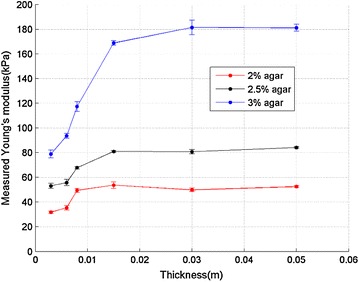
Relationship between the measured Young’s modulus and thickness (2, 2.5 and 3 % agar)

The typical SWE images of gelatin–agar phantoms with different thickness (3, 6, 8, 15, 30, 50 mm) are shown in Fig. 3. The color bar represents the Young’s modulus range. The colder color indicates lower Young’s modulus, while the warmer color indicates higher Young’s modulus. For the thin phantoms (3, 6 and 8 mm), the color distribution was homogeneous. For the thick phantoms (15, 30 and 50 mm), a narrow colder color zone presented right underneath the surface. This may be due to a boundary effect, which leads to depth dependent shear wave speed in the samples. For a thick sample, the large ROI captured this depth dependency and led to larger standard deviation within the ROI. For a thin sample, the depth range was very limited and thus did not show this effect. The mean values of measured Young’s modulus in all 18 phantoms are shown in Fig. 5. According to the experimental results, there was a nonlinear relationship between thickness and the measured Young’s modulus. When the thickness of phantom decreased to 15 mm or less, the measured Young’s modulus started to decrease. And this trend became more significant with further decrease of the phantom’s thickness. Obviously, sample thickness had an effect on the measured Young’s modulus, because the true material property should be independent of sample thickness as they are made from the same batch of agar-gelatin solution. In this study, the Young’s modulus measured in the 50 mm samples are considered “true” answer because the influence of thickness should be minimal for the 50 mm samples.

### FEM results and empirical model

**Fig. 6:**
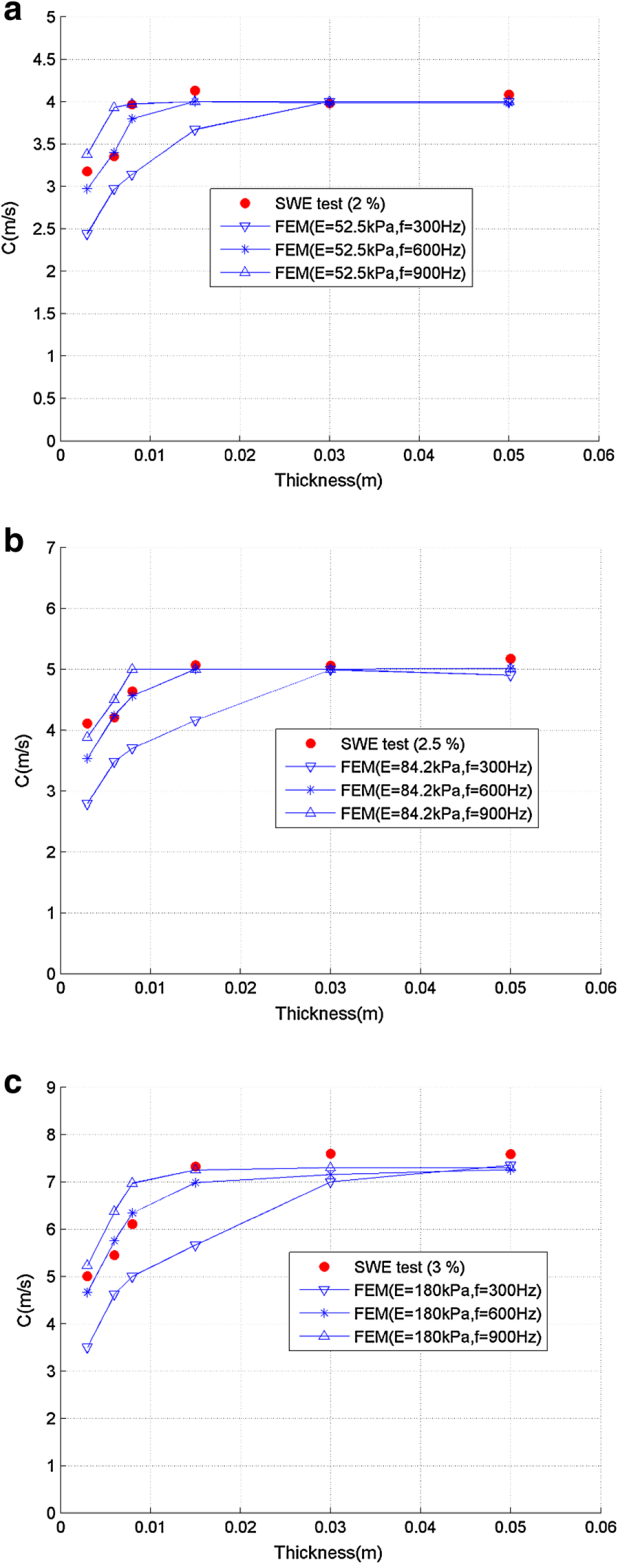
Comparison of shear wave speed between SWE tests and FEM results with different loading frequency (**a** 2 % agar, **b** 2.5 % agar, and **c** 3 % agar)

To investigate the propagation of shear wave in gelatin–agar samples, the finite element method was adopted with the following parameters: density 1050 kg/m^3^, Poisson’s ratio 0.499, thickness of plates 3/6/8/15/30/50 mm. Three different loading frequencies (300/600/900 Hz) were adopted in FEM to investigate the influence of loading frequency on wave speed. Figure 6 shows the comparison of shear wave speed between SWE tests and FEM results with different loading frequency. The shear wave speed from FEM also decreased with the decreasing thickness, and FEM results with frequency of 600 Hz matched best with SWE test results.

**Fig. 7:**
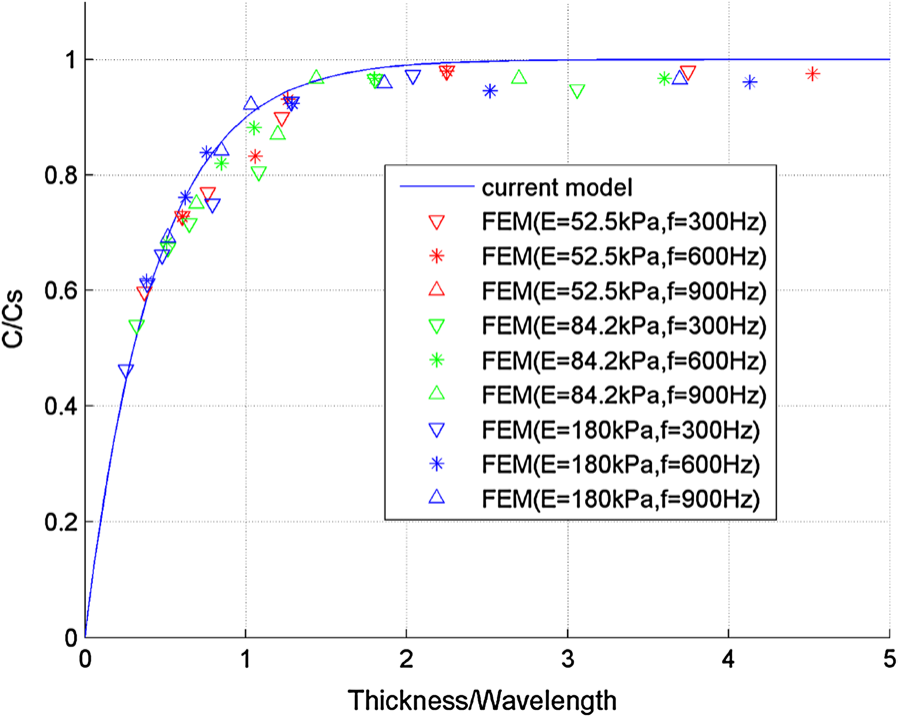
Relationship between non-dimension wave speed $\left( {\frac{C}{{C_{s} }}} \right)$ and non-dimension thickness $\left( {\frac{H}{C/f}} \right)$

Through above experiments and simulation, we confirmed that the shear wave speed decreased in thin layer structures, which can cause significant bias when one tries to use measured shear wave speed to deduce the Young’s modulus by Eq. (1). To develop a new empirical model to correct the bias in thin plate, dimensionless transformation for Fig. 6 was carried out as shown in Fig. 7, where the abscissa and ordinate are non-dimension thickness and non-dimension speed respectively. The non-dimension wave speed is the shear wave speed in thin plate divided by shear wave speed in an infinite field, and the non-dimension thickness is the sample thickness divided by wave length. According to non-dimension data in Fig. 7, a new empirical model was developed as below:

3 $$C_{s} = C/\left( {1 - \exp \left( { - 2.3\frac{\text{H}}{{{\text{C}}/{\text{f}}}}} \right)} \right)$$

where C and $C_{s} = \sqrt {G/\rho }$ are shear wave speed in thin plate and infinite field respectively. H and C/f are the thickness of the gelatin and wave length respectively, where f is the loading frequency. If desired, one can combined Eqs. (1) and (3) to generate the empirical formula relating Young’s modulus (instead of the shear wave speed) of the sample and the “true” results, as shown in Eq. (4).

4 $$E_{0} = E/\left[ {1 - { \exp }\left( { - 2.3\frac{H}{{\sqrt {E/3\rho } /f}}} \right)} \right]^{2}$$

where *E*_0_ and *E* is the corrected and appearant Young’s modulus respectively.

### Validation of the empirical formula

We calculated the “corrected” Young’s modulus by Eq. (4), using the “appearant” modulus measured by SWE and the sample thickness (which can be measured through B-mode imaging in practice) as inputs. Based on results in Fig. 6, the shear wave frequency was set at 600 Hz. The “corrected” Young’s modulus was compared with the “appearant” Young’s modulus in Table 1. The average values of the “corrected” Young’s modulus (52.5, 89.8 and 187 kPa) were very close to the “true” Young’s modulus (52.5, 84.2 and 182.2 kPa) of thick phantoms (50 mm).

**Table 1:** Comparison of appearant and corrected Young’s modulus

Thickness (mm)	Young’s modulus (kPa)						
	3	6	8	15	30	50	Mean value ± SE
2 % agar							
Appearant	31.8	35.4	49.5	53.7	49.9	52.5	
Corrected	59.9	42.2	56.2	54.4	49.9	52.5	52.5 ± 2.5
2.5 % agar							
Appearant	53.2	55.8	67.8	81.0	80.8	84.2	
Corrected	132.0	75.4	82.3	83.8	80.8	84.2	89.8 ± 8.5
3 % agar							
Appearant	78.9	93.6	117.3	168.9	181.5	182.2	
Corrected	249.2	153.4	167.8	190.8	183.1	181.2	187.6 ± 13.5

**Fig. 8:**
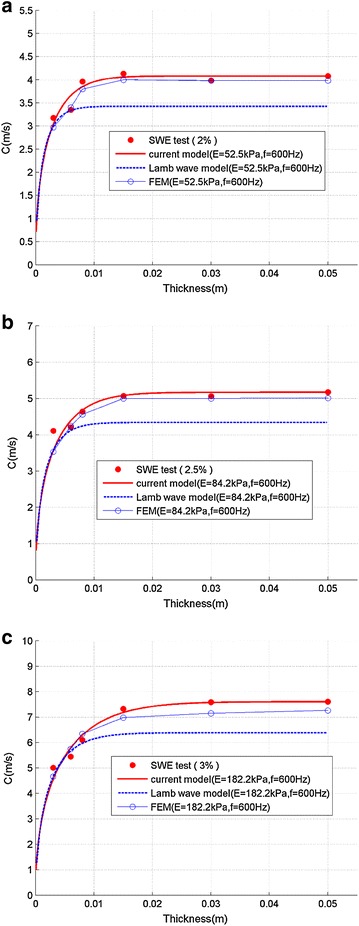
Comparison of shear wave speed between SWE, FEM, empirical model and Lamb wave model (**a** 2 % agar, **b** 2.5 % agar, and **c** 3 % agar)

Figure 8 shows the comparison of wave speed among SWE experiments, FEM, empirical model, and Lamb wave model. It can be seen from Fig. 8 that when the thickness of the plate sample is smaller than about 0.005 m, FEM result, Lamb wave model, and empirical equation all match well. But when the thickness increases, the Lamb wave model will underestimate the shear wave speed, whereas the empirical model shows good agreement with experimental results and FEM simulations for all samples of different thickness.

### Influence of Poisson’s ratio setting on FEM

**Fig. 9:**
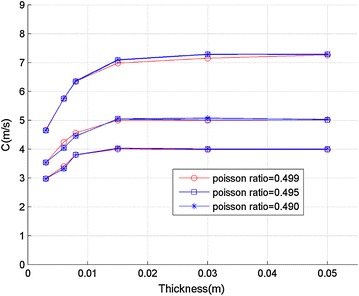
FEM results with different Poisson’s ratio

To clarity the influence of Poisson’s ratio setting on FEM, we used three different Poisson’s ratio (0.499, 0.495 and 0.490) and compared the results. As shown in Fig. 9, the FEM results with different Poisson’s ratio were close to each other, which suggests the Poisson’s ratio of 0.499 used in this study was adequate.

## Discussion

Our study confirmed that the appearant Young’s modulus measured by SWE is affected by sample thickness. Results from FEM and experiments indicates that shear wave speed decreased with thickness in thin layer samples, and the change was more significant when the thickness was less than 15 mm. When the thickness was larger than 30 mm, the SWE results of the phantoms did not change much. Lamb wave modeling agreed well with SWE experimental data for thin layer samples but had significant error for samples with thickness of 15 mm or above. When we used the empirical formula, the corrected Young’s modulus for the thin phantoms (3, 6, 8 and 15 mm) were much closer to that of the thick phantoms (30 and 50 mm).

This bias in SWE test is due to the slower guided wave propagation speed in thin layers. This guided wave propagation was first studied in Lamb (1917) and has been used for non-destructive testing applications. Isaaz (2002) first adopted Lamb wave equations of motion for estimation of myocardial viscoelasticity. Later on, Nenadic et al. (2011) extended Lamb wave modeling for shear wave experiments on urethane plate and porcine left ventricle free-wall myocardium. Although the Lamb wave model as shown in Eq. (2) can be used to account for geometric effects of the thin layer and solve for the correct material property, the function is relatively complex and the shear modulus is implicitly embedded in the equation. Therefore, it may be difficult for physicians to use this approach in daily clinical practice. In addition, our study demonstrates that Lamb wave model is inaccurate for thick layers. Here we proposed an alternative correction strategy based on a simple empirical formula as shown in Eq. (4), with which the true Young’s modulus can be conveniently calculated from this explicit equation using the sample thickness and the measured shear wave speed. Therefore, the proposed method may be more practical and “user-friendly”. In contrast with the Lamb wave correction approach, this empirical formula can be applied to a wide range of sample thickness.

This simple correction strategy may have significant clinical value in shear wave measurements in thin layer tissues such as myocardium, artery, bladder, and muscle. The thickness of target tissues can be measured by real-time B-mode imaging and used with the measured apparent shear wave speed to calculate the true material properties of the target tissues through the simple empirical formula proposed in this study. Although the study was performed using a linear array transducer, the simple empirical correction approach proposed here can be applied to shear wave measurements obtained with other transducers such as a curve array or a phase array. This correction is useful for reducing the confounding effect of tissue thickness and facilitates better comparison among different patients. The empirical formula is easy to use and can be applied to layer tissues of any thickness, and therefore would be suitable for clinical use.

There are several limitations of this study. First, the agar-gelatin samples prepared from the same solution may have small variations in stiffness across different samples. Large samples cure slower than smaller samples, possibly leading to different final stiffness. Therefore, the assumption that all samples had the same material properties may not necessary be true. This may also explain the discrepancy between experimental results and FEM/empirical-formula predictions. Second, the frequency of shear waves used in the experiments by the Aixplorer scanner was unknown to us, and was obtained by fitting FEM to experimental results. Future studies should investigate if the shear wave center frequency would change for SWE measurements using machines from different vendors. As a first order correction, one can still use the empirical formula and shear wave frequency of this study to provide a corrected measurement that is closer to the “true” answer. Third, the FEM simulation using a line load with four cycle of sinusoidal wave may be somewhat different from the ultrasound push beam used in Aixplorer to generate shear waves. This approximation may cause small discrepancy between FEM and SWE experimental results seen in the study. Fourth, the stiffness of samples used in this study were only calibrated with ultrasound shear wave elastography. An independent validation using mechanical testing would have strengthened the paper. Last, simulations and experiments were performed for samples surrounded by liquid. Therefore, the method may not be adequate for plates surrounded by soft tissues. In addition, the curvature of the plate, which may be important for structures such as vessels, was not considered in this study. Future studies should be conducted to investigate the impact of loading medium and structure curvature for more specific applications.

## Conclusion

In this study, we investigated the confounding effects of sample thickness on ultrasound shear wave elasticity measurements. Phantom studies demonstrated that the measured shear wave speed decreased with the decreasing sample thickness. This observation was confirmed by FEM simulation and Lamb wave analytical model. A simple empirical correction formula was proposed to account for the thickness effects, which was validated by SWE experiments. This simple correction strategy does not require complicated mathematical or engineering expertise, and thus may be useful for clinicians to compare shear wave measurements of thin layer tissue such as myocardium and bladder among different subjects.
